# Leishmaniosis in a cat with chronic diarrhea as the only clinical manifestation

**DOI:** 10.1111/jvim.16347

**Published:** 2022-01-17

**Authors:** María‐Dolores Tabar, Carolina Naranjo, Alexandra Dehesa, Mari‐Carmen Rodríguez

**Affiliations:** ^1^ Hospital Veterinario San Vicente‐Vetsum San Vicente del Raspeig Spain; ^2^ IDEXX Laboratories Barcelona Spain

**Keywords:** cat, diarrhea, *Leishmania* spp.

## Abstract

A 10‐year‐old male domestic shorthaired cat was presented with chronic diarrhea unresponsive to treatment. Laboratory testing identified hyperglobulinemia and mild nonregenerative anemia, and nongastrointestinal causes of diarrhea were ruled out. Gastrointestinal endoscopy and biopsy were performed and disclosed diffuse generalized granulomatous and lymphoplasmocytic inflammatory reaction in all segments of gastrointestinal tract evaluated, with numerous *Leishmania* spp. amastigotes within the cytoplasm of macrophages. The organism also was detected in spleen and bone marrow and *Leishmania* spp. serology was positive (immunofluorescence assay 1 : 160). A diagnosis of granulomatous enteritis secondary to leishmaniosis was made. Gastrointestinal signs resolved after treatment with allopurinol and a dietary supplement of nucleotides and active hexose‐correlated compounds (N‐AHCC), but seropositivity and gammopathy persisted 8 months later. The cat died of unrelated causes after an additional 3 months and permission for necropsy was not granted. Leishmaniosis as a cause of chronic diarrhea has not been reported previously in cats and should be considered in endemic areas in cats with chronic gastrointestinal signs.

**Abbreviations**
FeLV: feline leukemia virus
FIV: feline immunodeficiency virus
fPLI: feline pancreatic lipase
fTLI: feline trypsin‐like immunoreactivity
N‐AHCC: nucleotides and active hexose correlated compounds
RBC: red blood cells
T4: total thyroxine
UPC: urinary protein/creatinine ratio

## 1 INTRODUCTION

Leishmaniosis is an endemic disease in the Mediterranean area of Europe caused by the protozoan *Leishmania infantum* and transmitted by phlebotomine sand flies.^1^ Limited information is available on epidemiological and clinical aspects of *Leishmania* spp. infection in cats, but it appears to be an emerging disease in this species, more frequently reported in recent decades in endemic areas.^1^, ^2^ Clinical disease in cats is rare, and clinical signs usually reflect cutaneous and ocular disease, with or without visceral involvement.^1^, ^2^

Chronic diarrhea is an uncommon primary presentation of leishmaniosis (López M, Bertolani C, Sainz A, et al. Chronic diarrhea as a main clinical sign of canine leishmaniosis: 22 cases Proceedings of the 2020 ECVIM congress). In dogs, gastrointestinal signs most commonly are related to chronic renal disease.^3^ Similarly, vomiting and diarrhea have been reported sporadically in some cats with leishmaniosis, although not associated with primary gastrointestinal disease.^1^

## 2 CASE DESCRIPTION

A 10‐year‐old male neutered domestic shorthaired cat was presented for evaluation of chronic diarrhea. The cat lived in a multicat household and lived both indoors and outdoors. The cat had not received any vaccinations or treatment for intestinal parasites and ate a commercial maintenance dry food. On presentation, the main owner complaints were mild weight loss and small bowel diarrhea that started 4 weeks before presentation, and was not responsive to previous dietary changes and antibiotic treatment.

Mild scaly dermatitis and poor body condition (body condition score 3/9; 3.7 kg) were noted on physical examination. No abnormalities were detected on abdominal palpation and peripheral lymph nodes were slightly enlarged. Laboratory testing (CBC, serum biochemical profile and urinalysis) identified a mild nonregenerative anemia and hyperglobulinemia (Table 1). Mild proteinuria was detected. No parasites were found on fecal analyses (fresh saline fecal smear and zinc sulfate fecal flotation). Empirical treatment was started with fenbendazole (Panacur, Laboratorios Intervet, Salamanca; 50 mg/kg q24h for 5 days) and later with a commercial hydrolysed protein diet (Royal Canin Hypoallergenic, Royal Canin, Madrid, Spain) and probiotic (Fortiflora, Nestlé Purina Petcare España, Castellbisbal, Spain).

**TABLE 1:** Results of blood tests

Value	Day 0	Day 60	Day 90	Day 240	Reference range
Hematology					
Red blood cells	5.64 M/L	4.72 M/L		6.77 M/L	6.54‐12.20 M/L
PCV	31.0%	26.4%	25%	37.3%	30.3%‐52.3%
Reticulocytes	19.2 K/L	22.7 K/L		42.0 K/L	3.0‐50.0 K/L
White blood cells	4.32 K/L	3.01 K/L		6.58 K/L	2.87‐17.02 K/L
Platelets	170 K/L	180 K/L		185 K/L	151‐600 K/L
Biochemistry					
Total protein	8.6 g/dL	8.6 g/dL	8.11 g/dL	8.61 g/dL	5.7‐8.9 g/dL
Globulins	5.9 g/dL	6.1 g/dL	5.6 g/dL	5.63 g/dL	2.8‐5.1 g/dL
Albumin	2.7 g/dL	2.5 g/dL	2.51 g/dL	2.98 g/dL	2.3‐3.9 g/dL
Creatinine	1.5 mg/dL	1.4 mg/dL	1.4 mg/dL	1.7 mg/dL	0.8‐2.4 mg/dL
Blood urea nitrogen	22 mg/dL				16‐36 mg/dL
ALT	88 U/L				12‐130 U/L
ALKP	42 U/L				14‐111 U/L
Glucose	142 mg/dL				71‐159 mg/dL
Cloride	125 mmol/L				112‐129 mmol/L
Potassium	4.3 mmol/L				3.5‐5.8 mmol/L
Sodium	163 mmol/L				150‐165 mmol/L
UPC	0.45		0.22	0.23	
T4		2.6 μg/dL			0.8‐4.7 μg/dL
fTLI		20.3 μg/dL			12‐82 μg/dL
fPLI		2.9 μg/dL			<3.5 μg/dL
Cobalamin		>1000 ng/L			270‐1000 ng/L
Serum electrophoresis: gammaglobulins		3.45 g/dL (PG)	3.37 g/dL (PG)	3.33 g/dL (PG)	0.9‐2.5 g/dL
Serologies					
FIV/FeLV		Negative			
Li serology		1/160	1/160	1/160	<1/40

Abbreviations: ALKP, alkaline phosphatase; ALT, alanin aminotransferase; FeLV, feline leukemia virus; FIV, feline immunodeficiency virus; fPLI, feline pancreatic lipase; fTLI, feline trypsin‐like immunoreactivity; Li, *Leishmania infantum*; PG, polyclonal gammopathy; T4, serum total thyroxine; UPC, urinary protein/creatinine ratio.

**FIGURE 1:**
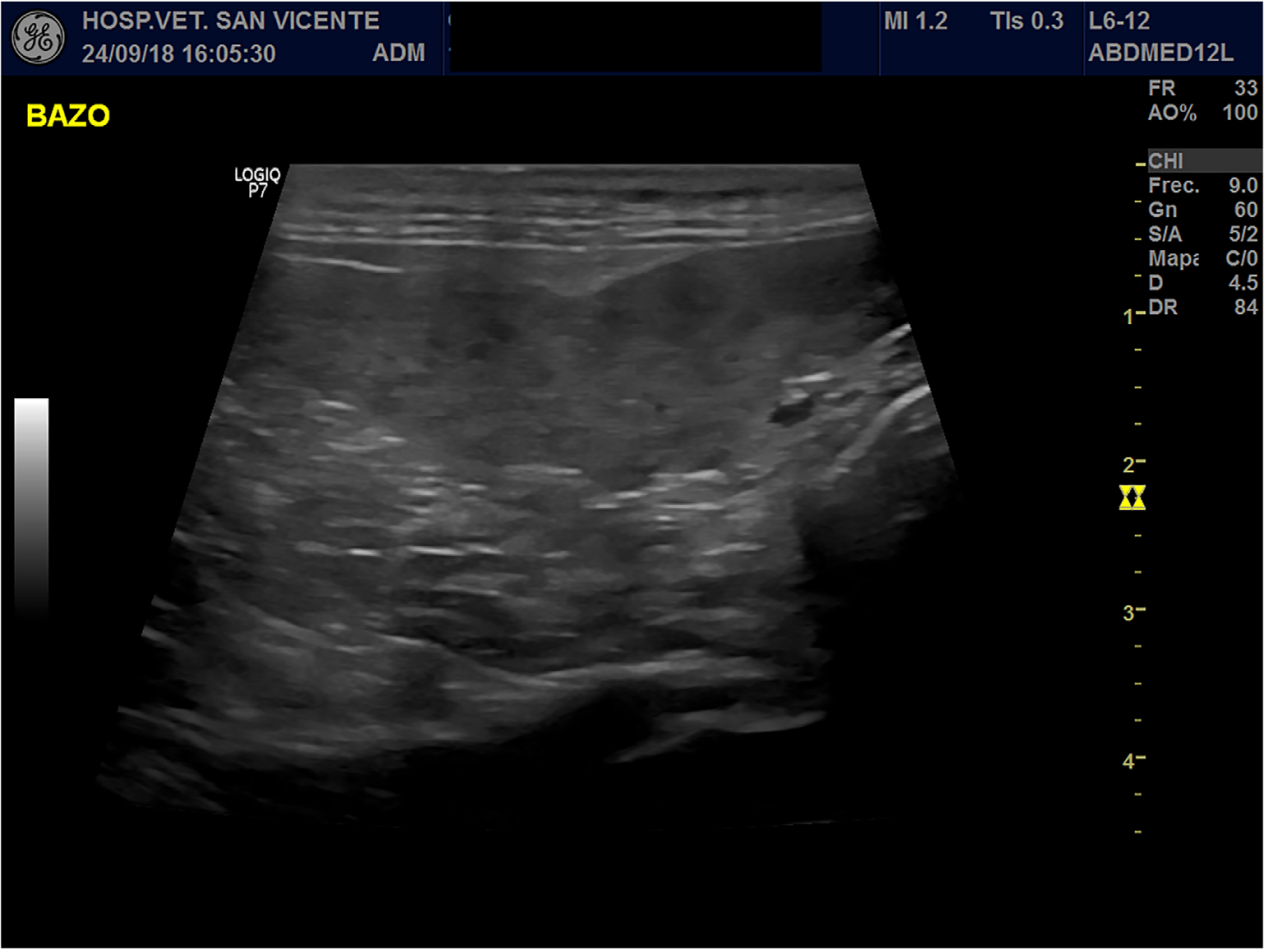
Honeycomb appearance of spleen on ultrasonography

Two months later, the cat weighted 4.18 kg. Initially, partial improvement in clinical signs occurred, but diarrhea persisted. At this time hematology disclosed worsening of anemia and globulin concentration was persistently increased (Table 1). Serum total thyroxine concentration (T4), feline trypsin‐like immunoreactivity (fTLI) and feline pancreatic lipase (fPLI) concentration were within normal limits, and serum cobalamin concentration was increased (Table 1). Tests for feline immunodeficiency virus (FIV) and feline leukemia virus (FeLV) were negative (SNAP Combo Plus IDEXX, Hoofddorp, Netherlands). Abdominal ultrasound examination identified splenomegaly with a mottled or honeycomb appearance (Figure 1) and slight increase in thickness of the duodenum (2.6 mm). Fine needle aspiration of spleen and lymph node were performed and *Leishmania* spp. amastigotes were detected on cytology. Bone marrow cytology also was performed, and *Leishmania* spp. amastigotes were detected both free and in the cytoplasm of macrophages.

Anti‐*Leishmania* spp. antibodies were detected by immunofluorescence assay at a titer of 1 : 160 (Inmunovet, Barcelona, Spain), and serum electrophoresis indicated a mild increase in alpha‐2 globulins and a polyclonal gammopathy.

Leishmaniosis was diagnosed, but gastrointestinal endoscopy and biopsy were recommended to detect any concurrent primary gastrointestinal disease that could explain the chronic diarrhea (eg, gastrointestinal lymphoma, inflammatory bowel disease) and that could affect clinical management.

The gastrointestinal tract was examined using a flexible video endoscope (Olympus GIF‐160, Rungis, France) after a cleansing laxative agent (Solución evacuante Bohm, Laboratorios BOHM, Fuenlabrada, Spain) and warm water enemas. The cat was examined under general anesthesia, using propofol for induction and isoflurane for maintenance. Gastroscopy showed signs consistent with moderate nonspecific gastritis. The duodenum appeared edematous with increased diffuse granularity and was friable upon biopsy. Ileocolonoscopy disclosed a diffusely hyperemic mucosa, with several focal erosions. Samples were taken from all gastrointestinal segments, fixed in formalin, and submitted for histopathological analysis.

**FIGURE 2:**
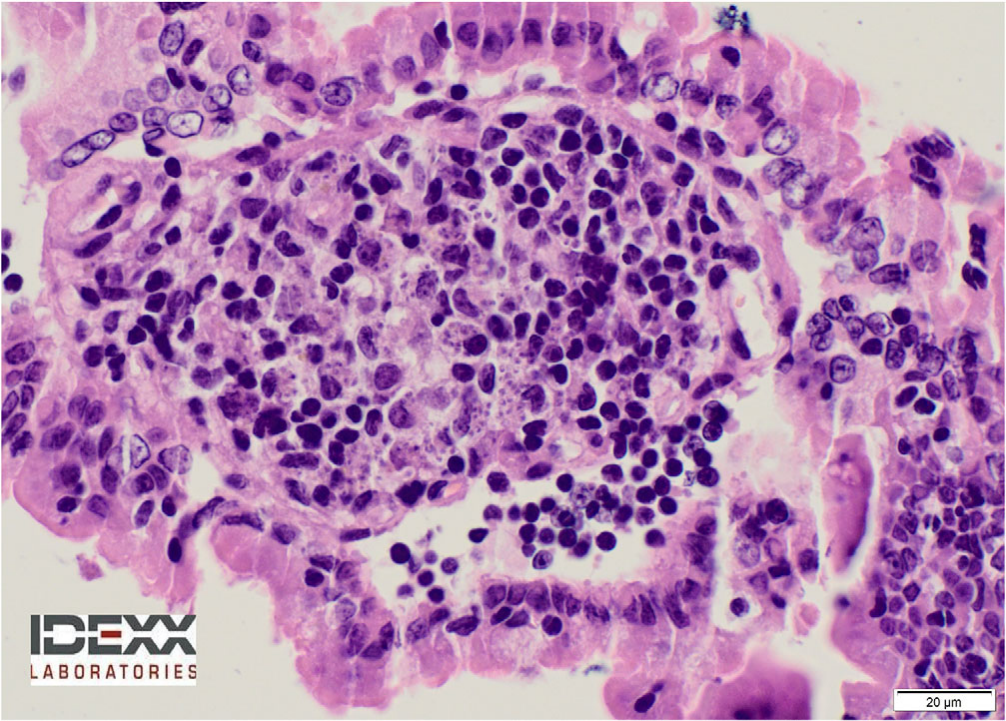
Duodenum. Granulomatous and lymphoplasmacytic inflammation in the lamina propria of the mucosa, with intracellular parasite forms consistent with *Leishmania* spp. amastigotes. Hematoxylin and eosin

**FIGURE 3:**
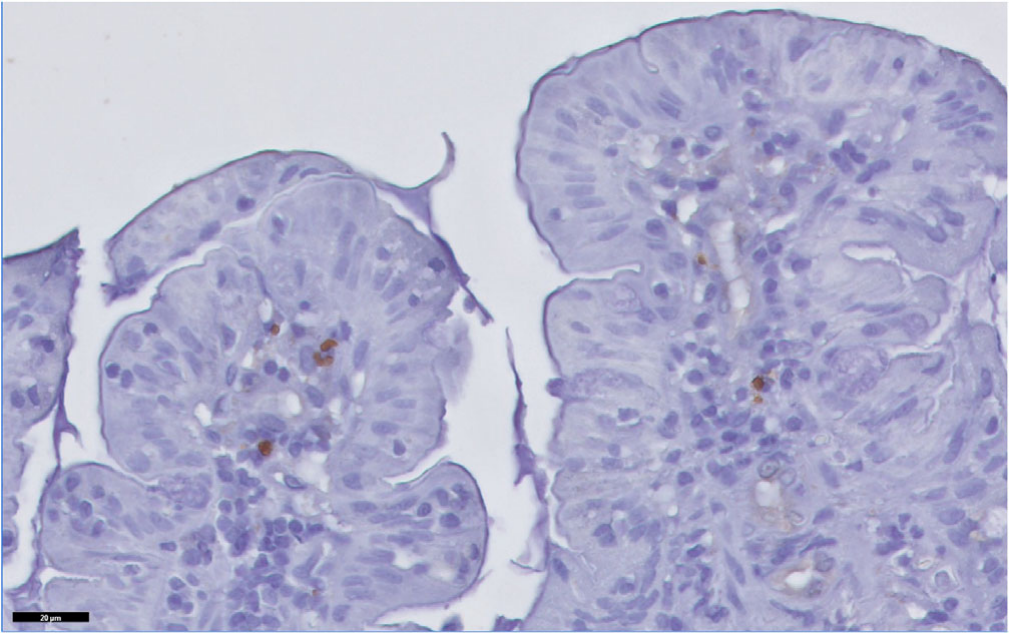
Immunohistochemistry confirmed the presence of intracytoplasmic *Leishmania* spp. amastigotes within macrophages. Immunohistochemistry (immunoperoxidase)

Histologically, a diffuse generalized granulomatous and lymphoplasmocytic inflammatory reaction was found within the lamina propria of the mucosa, more severe in the duodenum, but also in the stomach, ileum, and cecum. Intracellular parasitic forms morphologically consistent with *Leishmania* spp. amastigotes were seen within the cytoplasm of numerous macrophages throughout the gastrointestinal tract (Figure 2). The presence of *Leishmania* spp. was confirmed by immunohistochemical staining (Figure 3). A diagnosis of granulomatous gastroenteritis caused by *Leishmania* spp. infestation was made.

Treatment was started with allopurinol (Zyloric, Faes Farma S.A., Lamiaco [Lejona], Vizcaya; 10 mg/kg q12h) and a dietary supplement of nucleotides and active hexose‐correlated compounds (Impromune, Bioiberica, Barcelona; N‐AHCC; ½ tablet PO q24h). Subcutaneous treatment with meglumine antimoniate was not attempted because of the fractious nature of the cat. One month later diarrhea had resolved, weight had increased to 5 kg, urine protein : creatinine ratio (UPC) had normalized, but gamma globulins were still increased. Eight months after diagnosis, the cat continued on medication, and was healthy without gastrointestinal signs. No alterations in hematology or biochemistry were observed except for persistent gammopathy (Table 1), but amastigotes of *Leishmania* spp. still were found on cytology of a splenic aspirate and antibody titers had not decreased (1 : 160). The cat was lost for follow‐up until 3 months later when it was admitted to the emergency service with clinical signs of sepsis, likely related to an abscess in the right forelimb. Unfortunately, the cat died shortly after hospitalization and necropsy was not allowed.

## 3 DISCUSSION

Primary gastrointestinal disease associated with leishmaniosis is not commonly reported, even in dogs. In dogs with leishmaniosis, clinical signs such as vomiting and diarrhea more frequently are secondary to renal disease, but they also have been linked to hepatopathy, pancreatitis or gastrointestinal inflammation.^3^, ^4^, ^5^, ^6^ Asymptomatic colitis has been reported in dogs with natural infection,^7^ and other investigators have found parasites in all gastrointestinal segments with a higher parasite load in the cecum and colon.^8^ As can occur in endemic areas where *Leishmania* spp. can be detected in the skin or other organs of healthy dogs,^9^ those studies suggested that *Leishmania* spp. can be present in the intestine without lesions or clinical signs, possibly by taking advantage of the intestinal immunoregulatory response.^8^ Isolated reports however also have linked leishmaniosis to chronic gastritis and enterocolitis in some dogs.^10^, ^11^ Chronic diarrhea has not been previously reported as a primary presentation of *Leishmania* spp. infection in a cat. Vomiting and diarrhea are included in the lists of clinical findings that can be detected in leishmaniosis in cats, but they are present in <25% of cases and usually reflect extragastrointestinal disease.^1^ One case report described a cat presented with acute jaundice and vomiting that had *Leishmania* spp. amastigotes detected at necropsy in several different abdominal viscera, including stomach and large intestine.^12^

Findings from endoscopy and histopathology in the cat of the present report were similar to those reported in dogs, where patches of hyperemic, edematous, irregular and mildly erosive colonic mucosa are commonly encountered, and the most frequent inflammatory pattern is pyogranulomatous.^7^ Although in our case the inflammation was more severe in the small intestine, the entire gastrointestinal tract was affected, and the inflammation was primarily granulomatous and lymphoplasmocytic.

Underlying immunological dysfunction may allow active multiplication of the parasite and clinical disease in cats, as has been suggested in some reports that have found leishmaniosis more frequently in immunosuppressed cats or those with coexisting diseases.^13^ Possible concurrent gastrointestinal disease in the cat could have acted as an immunosuppressive factor that favored *Leishmania* spp. infection, but a gastrointestinal condition (independent of leishmaniosis) or any other condition that suggested impaired immunocompetence was not identified.

When other causes have been ruled out by diagnostic testing and treatment trials in cats with chronic diarrhea, biopsy is the next step to confirm the presence of idiopathic inflammatory bowel disease or neoplasia (mainly lymphoma). However, in some cases in which there are financial constraints, comorbidities that can preclude safe anesthesia, or other findings that suggest an immune‐mediated etiology (eg, triaditis), immunosuppressive treatment sometimes is attempted without a definitive diagnosis. This approach can be harmful if leishmaniosis is the cause of the gastrointestinal inflammation. Ideally, biopsy should be performed before immunosuppressive treatment is instituted in cats with chronic diarrhea, and this approach must be emphasized in cats living in areas endemic for leishmaniosis.

Some clinicopathological abnormalities can be suggestive of leishmaniosis. Nonregenerative anemia and increased globulin concentration were detected in the cat described here. Hypergammaglobulinemia was present in a recent case series in 87.5% of affected cats, with hyperproteinemia only present in 12.5%.^13^ Nonetheless, polyclonal gammopathy occurs in other infectious and inflammatory conditions and is not specific for leishmaniosis. Moreover, a normocytic normochromic (nonregenerative) anemia is also the most common hematological alteration reported in cats with leishmaniosis.^2^, ^3^, ^4^, ^5^, ^6^, ^7^, ^8^, ^9^, ^10^, ^11^, ^12^, ^13^

The honeycomb pattern observed on splenic ultrasonography in this cat is notable. Previous studies more frequently correlate this pattern with lymphoid hyperplasia and less commonly with neoplasia (mainly lymphoma), extramedullary hematopoiesis and splenitis.^14^ However, recently it also has been linked to leishmaniosis (Carrasco M, Carrillo S, Tabar MD. Honeycomb spleen pattern in dogs and cats with leishmaniosis. Proceedings of the 2021 ECVIM). Therefore, in endemic areas, leishmaniosis should be included in the differential diagnosis of this ultrasonographic finding, likely because leishmaniosis can cause splenitis, and splenic cytology can be helpful in the diagnosis.

Limited information is available about treatment and prognosis of leishmaniosis in cats. Standardized treatment is lacking, and treatment is instituted empirically using the drugs most commonly prescribed to affected dogs, including allopurinol as monotherapy or in combination with meglumine antimoniate as the most frequently used regimens.^1^, ^2^, ^13^, ^15^, ^16^ Our cat showed resolution of diarrhea with allopurinol and N‐AHCC. Because of the difficulty in using injectable drugs in this cat, N‐AHCC was added with the aim to complement treatment, with the advantage that it is already registered for use in cats. Although N‐AHCC has been used previously by the authors in other cats with good results (Dominguez‐Ruiz M, Hernández‐Rodríguez J, Tabar‐Rodriguez MD. Leishmaniosis en un gato con pancitopenia. Proceedings South European Veterinary Conference, November 2019, Madrid, Spain), and has been reported in another case,^16^ controlled studies concerning its effectiveness in cats with leishmaniosis are lacking. However, studies in dogs point to its efficacy in the treatment of leishmaniosis to prevent disease progression.^17^, ^18^ Moreover, an exogenous supply of nucleotides is essential for immune competence, intestinal development, and recovery, especially when an increased demand for nucleic acid synthesis exists, such as in situations when rapidly proliferating tissues (eg, intestine, immune system) fail to achieve their nucleotides needs. Therefore, N‐AHCC can be a potentially useful treatment option in gastrointestinal disease related to leishmaniosis because they have been reported to increase T‐helper 1 (Th1) cell responses and exert immunomodulatory effects on intestinal epithelial cells and macrophages.^19^

This report describes a novel clinical presentation of leishmaniosis in a cat with primary gastrointestinal signs. Because clinical leishmaniosis is rare in cats and these clinical signs are not commonly recognized features of the disease, it may be underdiagnosed. Therefore, in endemic areas, leishmaniosis should be considered in cats with chronic gastrointestinal signs, especially diarrhea.

## CONFLICT OF INTEREST DECLARATION

Authors declare no conflict of interest.

## OFF‐LABEL ANTIMICROBIAL DECLARATION

Authors declare no off‐label use of antimicrobials.

## INSTITUTIONAL ANIMAL CARE AND USE COMMITTEE (IACUC) OR OTHER APPROVAL DECLARATION

Use of medical information from medical record was approved by Hospital Veterinario San Vicente‐Vetsum and client consented to the use of such information.

## HUMAN ETHICS APPROVAL DECLARATION

Authors declare human ethics approval was not needed for this study.
